# Improvement of motion artifacts using dynamic whole-body ^18^F-FDG PET/CT imaging

**DOI:** 10.1007/s11604-023-01513-z

**Published:** 2023-12-14

**Authors:** Tomohito Kaji, Kouji Osanai, Atsushi Takahashi, Atsushi Kinoshita, Daiki Satoh, Tomoaki Nakata, Nagara Tamaki

**Affiliations:** 1 Department of Radiology, Division of Nuclear Medicine and PET Center, Hakodate Goryokaku Hospital, 38-3 Goryokaku-Cho, Hakodate, Hokkaido 040-8611 Japan; 2 Department of Radiology, Kyoto Prefectural University of Medicine, 465 Kajii-Cho, Kawaramachi-Hirokoji, Kamigyo-Ku, Kyoto, 602-8566 Japan; 3 Kyoto College of Medical Science, Oyama-Higashi, Sonobe, Nantan, Kyoto, 622-0041 Japan

**Keywords:** PET/computed tomography, FDG, Dynamic whole-body imaging, Body motion artifact

## Abstract

**Purpose:**

Serial dynamic whole-body PET imaging is valuable for assessing serial changes in tracer uptake. The purpose of this study was to evaluate the improvement of motion artifacts in patients using serial dynamic whole-body ^18^F-fluorodeoxyglyucose (FDG) PET/CT imaging.

**Materials and methods:**

In 797 consecutive patients, serial 3-min dynamic whole-body FDG PET imaging was performed seven times, at 60 or 90 min after FDG administration. In cases with large body motion during imaging, we tried to improve the images by summing the images before body motion. An image quality study was performed on another 50 patients without obvious body motion using the same acquisition mode.

**Results:**

Obvious body movement was observed in 106 of 797 cases (13.3%), and severe motion artifacts which interfered image interpretation were observed in 18 (2.3%). In these 18 cases, summation of the images before the body movement enabled us to obtain images that excluded the effect of the body motion. In the visual evaluation of the image quality in another 50 patients studied, acceptable image quality was obtained when 2 or more times the serial 3-min image data were added.

**Conclusion:**

Serial dynamic whole-body FDG PET imaging can minimize body motion artifacts by summation of the images before the body motion. Such serial dynamic study may be a choice for PET imaging to eliminate motion artifacts.

## Introduction

Positron emission tomography (PET)/computed tomography (CT) with ^18^F-fluorodeoxyglucose (FDG) is widely used for tumor detection, initial staging, evaluation of the treatment response, detection of recurrence and estimation of prognosis [1–5].

In daily clinical practice, however, adequate-quality images may not be obtained for image interpretation due to movements of the patient's body during imaging. Body motion during imaging can cause a large misalignment between PET and CT images and a significant loss of image quality. When the patient is unable to remain at rest until the end of imaging, the PET acquisition may be considered an incomplete study [6].

Recently some new PET systems allow for serial whole-body dynamic imaging using continuous bed motion [7–9]. At our hospital, we perform 7 repetitions of 3-min whole-body imaging for almost all subjects, and the data from the 7 repetitions are added together to produce diagnostic PET images for general interpretation [10, 11].

The purpose of this study is to investigate whether, in cases with large-scale body motion during imaging, it is possible to exclude the effect of that motion by summation of the prior image data. We will examine how many times such addition is necessary to obtain sufficient image quality to allow for diagnostic interpretation.

## Materials and methods

All procedures performed in studies involving human participants were in accordance with the ethical standards of the institutional and national research committee and with the 1964 Helsinki Declaration and its later amendments or comparable ethical standards. No animals were used in the present study, by any author. Based on the Ethical Guidelines for Medical and Health Research Involving Human Subjects of the Ministry of Health, Labour and Welfare, a waiver of informed consent for the retrospective analyses of the anonymized clinical data in this study was obtained from the Institutional Review Board of Hakodate Goryokaku Hospital, Hokkaido, Japan.

### Patients

Serial dynamic whole-body FDG PET acquisition was performed routinely at our hospital to investigate malignancy. The cases where the existence of metastatic lesions around pelvic organs was suspected were excluded from dynamic imaging to avoid the influence of gradual changes in bladder size during dynamic PET acquisition. Accordingly, serial dynamic whole-body imaging was performed in 797 patients from February 25th to September 17th in 2019. Gender of all 797 subjects was 464 males and 333 females, age was 68.0 ± 11.9, the blood sugar level was 110.9 ± 25.3 mg/dl. The breakdown of these case was 300 lung and pleural and thymus cancer, 122 lymphoma and myeloma, 99 cancer screening, 75 breast cancer, 64 upper gastro intestinal cancer, 35 head and neck cancer, 34 liver and biliary tract and pancreas cancer, 27 colon and rectal cancer, 15 unknown cancer, 12 kidney and urinary tract and prostate cancer, 7 inflammatory diseases, 5 skin cancer and melanoma, 2 gynecological cancers. Those who was apparently looked impossible to stay rested, for example the patient with dementia, were sedated in advance. In patients who moved, we summed the images prior to the movement.

We evaluated another 50 patients to know the number of image summation required to get acceptable or enough images for the purpose of image interpretation. Serial dynamic whole-body imaging was performed from June 15th to 26th 2020. Gender of all 50 subjects is 28 males and 22 females, age is 65.9 ± 14.5, the blood sugar level is 110.3 ± 14.9.

### FDG PET/CT protocols

FDG 3.0 MBq/kg body weight was administered intravenously after patients fasted for more than 5 h, and PET/CT imaging was performed in the supine position after 60 or 90 min of rest, using a PET/CT system. (Biograph Horizon, Siemens Medical Solutions). A low-dose CT scan was acquired for PET attenuation correction, anatomical information and image fusion. Dynamic whole-body FDG PET imaging was then performed 7 times with continuous bed motion at varying speeds (7.0 mm/s from the head to the chest, 4.2 mm/s for the upper abdomen, 7.0 mm/s from the lower abdomen to the pelvis) to acquire each whole-body phase in about 3 min in list mode. For the purpose of routine image interpretation, seven dynamic phases were summed in a reconstruction. The dynamic whole-body PET images and the summed whole-body PET image were reconstructed using attenuation-corrected ordinary Poisson three-dimensional ordered subset expectation maximization (3D-OSEM: subset 10, iteration 4) reconstruction with a 4-mm post-reconstruction Gaussian filter, using a 180 × 180-pixel matrix. Time of Flight (TOF) and Point Spread Function (PSF) were both used.

### Image summation and assessment

For the purpose of routine image interpretation, all dynamic phases were summed in a reconstruction. One board-certificated nuclear medicine physician and four radiologists specializing in nuclear medicine visually evaluated the PET and CT misregistrations on the fusion images and determined that the cases that caused problems in identifying the site of accumulation or errors in attenuation correction were those with particularly large body movements. The final decision was made by a nuclear medicine specialist. In the case of large body movements, each of the 7 serial imaging data was individually reconstructed to confirm the timing of the movement, and only the data obtained prior to the movement were summed to produce the diagnostic PET image. For image quality evaluation, PET images were created using the data from the first dynamic imaging and the summed data from the 1st to the 2nd, 3rd, 4th, 5th, 6th and 7th dynamic imaging. In each case, image summation was performed before reconstruction process.

One board-certificated nuclear medicine physician and 4 radiologists specializing in nuclear medicine visually assessed image quality of the summed images to score as follows: 3, good quality for interpretation; 2, acceptable quality for interpretation; and 1, difficult to read the image.

## Results

Apparent body movement was observed in 106 of 797 cases (13.3%). Among these, 18 cases (2.3%, 12 males and 6 females, age is 71.1 ± 9.5) showed severe motion artifacts that interfered with image interpretation.

**Fig. 1:**
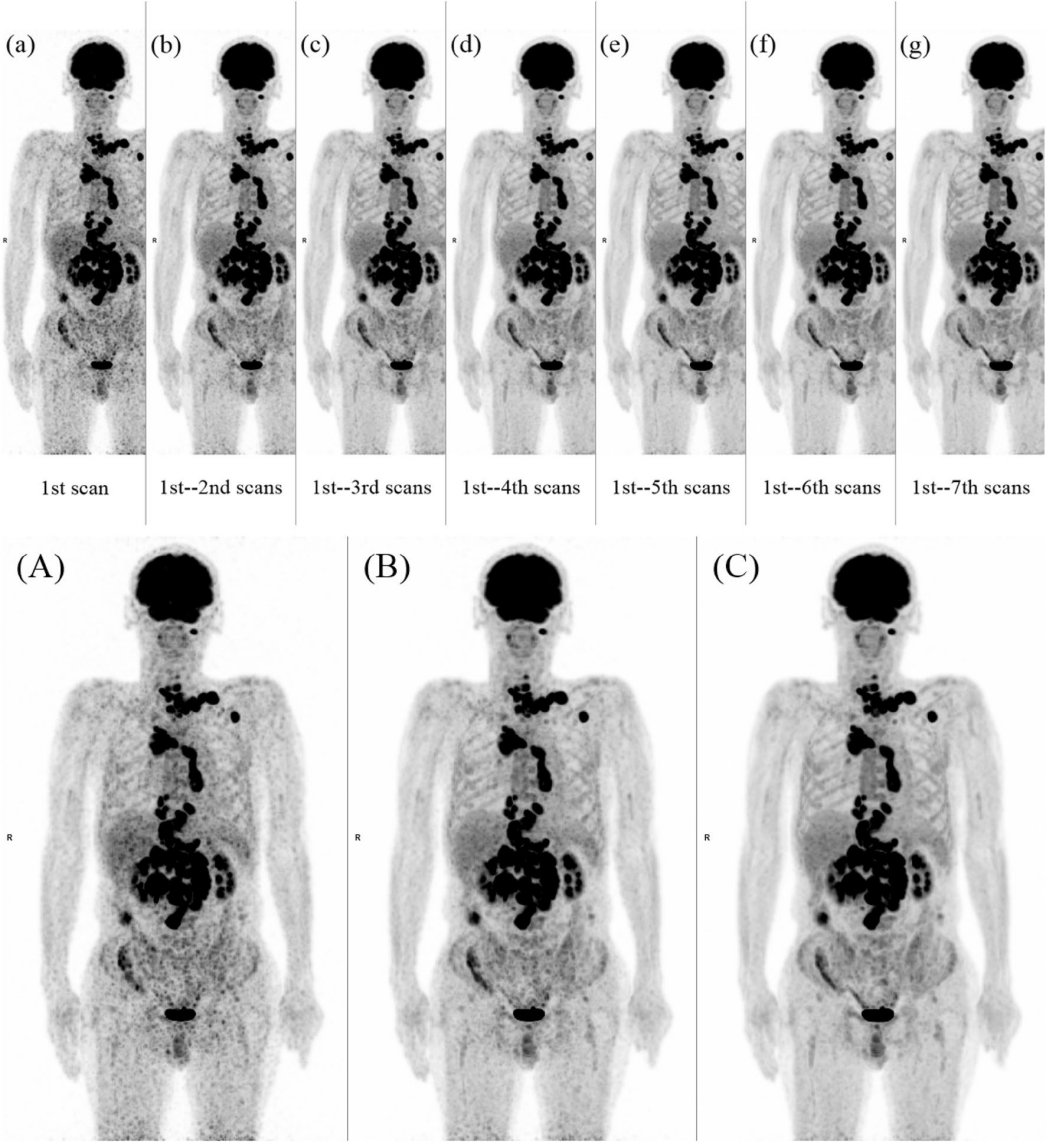
One serial whole-body dynamic ^18^F-FDG PET image a and six summed images **b**–**g** of a pretreatment malignant lymphoma patient. The image quality improves as the number of the summed images increases. Images **A**–**C** were enlarged image **a**, **b**, **d** as samples of quality score 1, 2 and 3

Three of the 18 cases were patients with reduced cognitive function for various reasons and another three were using analgesics to allow for the examination; however, the other 12 subjects had no obvious reason for not maintaining an immobile position. One case moved her torso, 8 cases moved their forearms, 5 cases rotated their head and the remaining 4 cases both rotated their head and moved their forearms (Fig. 1).

**Fig. 2:**
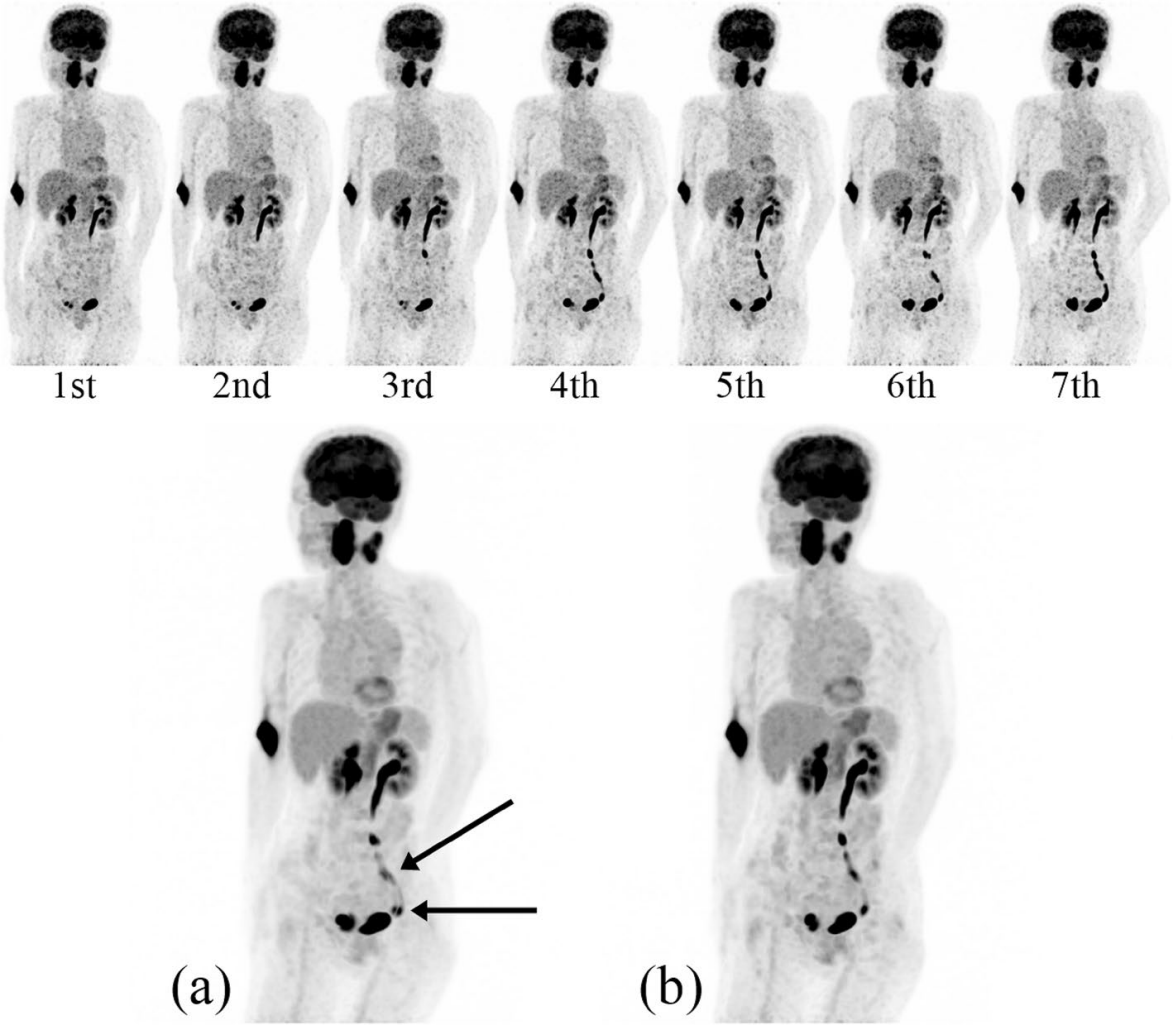
Serial whole-body ^18^F-FDG PET images of a patient suffering from oropharyngeal cancer. Activity in the ureters is seen on these seven serial whole-body dynamic ^18^F-FDG PET images. In particular, a motion artefact is noted on the 6th scan when she moved her pelvis slightly. Summation of 1st**–**7th scans **a** demonstrates an image artifact like double ureter (arrows). Summation of 1st**–**5th scans **b** shows the left kidney and single ureter

**Fig. 3:**
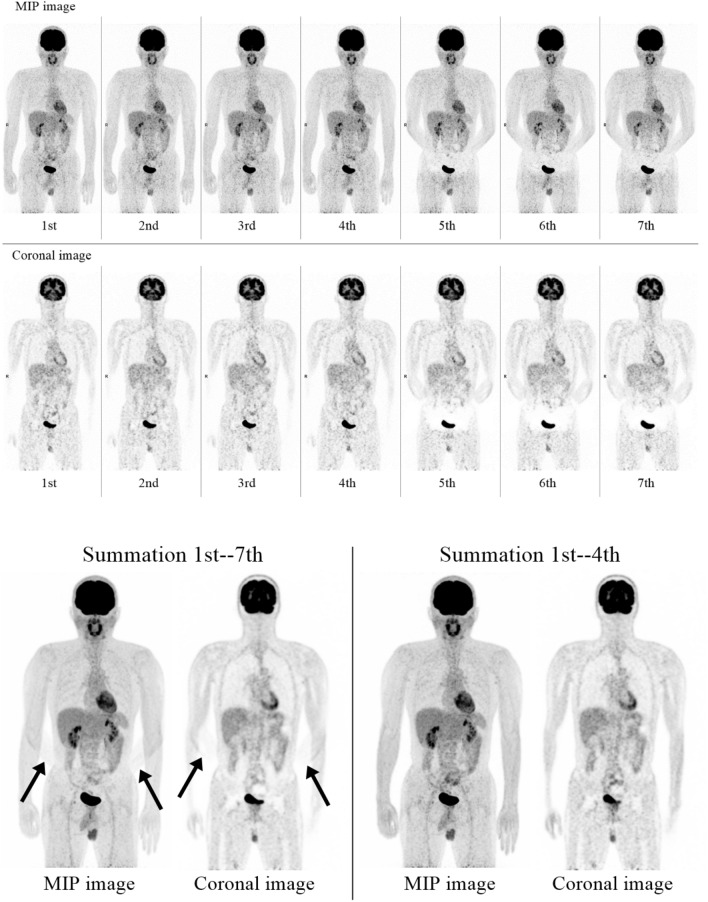
**1**. Serial whole-body ^18^F-FDG PET images of a case undergoing cancer screening. The patient moved his forearms on the 5th scan, and error of attenuation correction in the lower abdomen occurred in 5th to 7th scan. **2** Summation of 1st**–**7th scans (left) shows extra two forearms (arrows) and an error of attenuation correction in the lower abdomen especially on the coronal image. The summation of 1st**–**4th scans (right) shows only two forearms, and the attenuation correction is calculated correctly

**Fig. 4:**
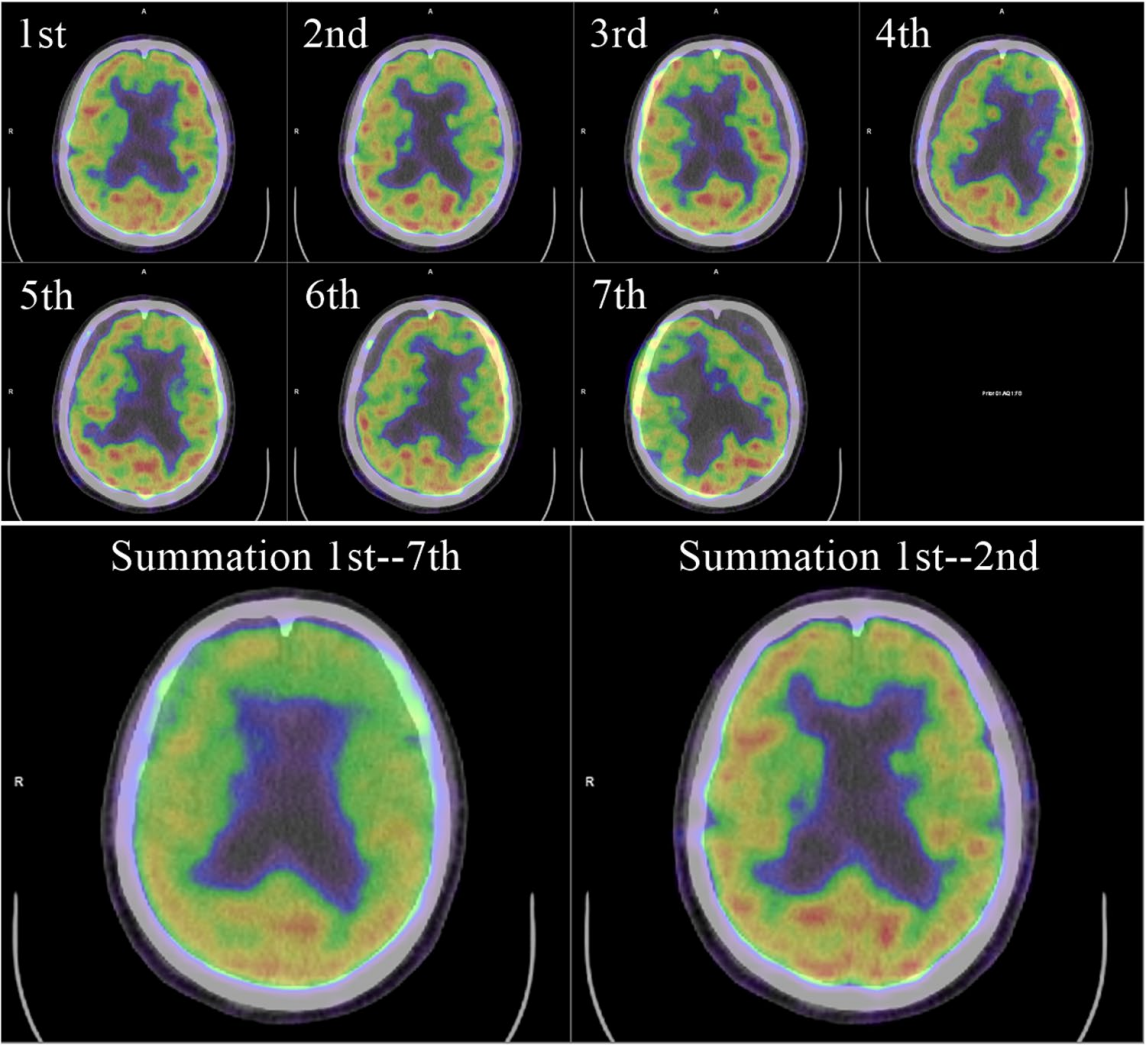
Axial images of serial whole-body dynamic^18^F-FDG PET/CT in a case undergoing cancer screening. The patient slightly rotated his head on    the 3rd  scan and largely rotated it on the 4th and 7th   scan (upper and middle row). Although the contour of cerebral construction is unclear and apparent laterality and anteroposteriority of cerebral glucose metabolism due to error of attenuation correction is shown in the image of 1st–7th summation (lower left), the summation of the 1st–2nd images shows the cerebrum clearly and error of attenuation correction is corrected (lower right)

Among all 18 cases with large body motion, serial dynamic images identified large body motion on the 3rd imaging in one patient and after the 3rd PET imaging in the remaining 17 cases. In these 18 cases, the serial dynamic images only before the body motion were selected for summing up for further analysis. Accordingly, misalignment between PET and CT and attenuation correction artifacts were eliminated (Figs. 2, 3, 4).

In the visual evaluation of image quality, the average score of the first dynamic image was 1.02 ± 0.14, and of summed images from the 1st to the 2nd, 3rd, 4th and 5th or later dynamic images were 1.74 ± 0.53, 2.52 ± 0.55, 2.94 ± 0.24, 3.0, respectively (Table 1). Thus, the image quality of only the first dynamic image was inadequate for clinical diagnosis. The image quality of the summed two images was partially adequate and that of the summed three or more images were interpretable for diagnosis.

**Table 1:** Average scores of the visual assessment of summed images by one nuclear medicine physician and 4 radiologists specializing in nuclear medicine

1st scan	1st–2nd scan	1st–3rd scan	1st–4th scan	1st–5th scan	1st–6th scan	1st–7th scan
1.02 ± 0.14	1.74 ± 0.53	2.52 ± 0.55	2.94 ± 0.24	3.0	3.0	3.0

The first dynamic images and the summed images are scored as follows: 3, good quality for interpretation; 2, acceptable quality for interpretation; and 1, difficult to read the image

## Discussion

Our results indicate that body motion is occasionally observed during PET/CT studies. Indeed, some patients showed severe motion artifacts. But such motion artifacts were solved when only the images before the body movement were added for image interpretation. Thus, serial whole-body dynamic acquisition seems to be valuable for minimizing motion artifacts.

PET/CT studies are often used for those who may have difficulties in understanding and cooperating with the examination, such as cognitively impaired patients and children or difficulty maintaining a position for a long period of time due to pain. It may be necessary to redo the imaging or postpone the examination due to body movements during the examination. If the examination is finished, large body motion during the imaging procedure may cause severe motion artifacts [12–15]. Some suggestion about minimize motion artifact may be especially important for patients who may be likely to move during a PET study. However, 12 of the 18 cases showing large body motion could be remains still. Thus, such serial dynamic study may be a choice for PET imaging to eliminate motion artifacts as much as possible.

Recent PET/CT systems have high sensitivity and high spatial resolution, achieved using smaller, high-performance PET detectors and advanced reconstruction software that enables shorter acquisition times compared with earlier systems. In addition, continuous-bed-motion mode, which is available for whole-body PET imaging with some PET cameras, provides better uniformity and reproducibility throughout the field of view compared with step-and-shoot acquisition mode [7–9]. However, it still requires ten or more minutes for whole-body FDG PET imaging even if using the latest PET/CT systems. Theoretically, SUV is not dependent on imaging time. But actually, SUVmax and MTV calculation is dependent on image quality. Therefore, higher FDG dose and longer imaging time may improve image quality and increase the accuracy of SUV and MTV calculations. In addition, SUV may change on imaging time point after FDG administration.

Serial whole-body dynamic imaging is useful for identifying physiological uptake in motion, such as the GI tract and ureters [11,15,16]. In this study, since most large body movements were observed after the midpoint of PET imaging, we were able to exclude the effect of body motion by summing together the images before the body motion. However, if body motions are observed at an early point, it may be necessary to add images after the body movements.

Our image quality assessment showed interpretable image quality after summation of the 1st to 2nd or 3rd scan (6 or 9 min) for whole-body imaging. In 14 of the 50 cases in which the image quality of the summed two images was examined, all of the evaluators judged the images to be diagnostic, and in 39 of these 50 cases the majority of the evaluators, three out of five, judged the images to be diagnostic. Although not all cases can be diagnosed with summed two images, if two scans can be performed, there is a high possibility that a repeat examination can be avoided. Therefore, even in cases where large body movements were observed during imaging, misalignment between PET and CT was eliminated, and the attenuation correction artifacts could be solved when the patient had been resting for about 6 or 9 min before the body motion. These results of image quality may be somewhat altered depending on the equipment sensitivity, FDG dose and many other conditions [7–9]. Fortunately, diagnostic image quality was obtained by deleting images after body movement in the cases that showed large movement in this study, but retesting may be necessary if sufficient image quality cannot be obtained. It should be noted that this may not be valid in all cases.

This procedure is easy to perform routinely, and we currently perform dynamic imaging in almost all cases. Total acquisition time of 21 min (7 times for 3 min each) is considered to be within the range for routine clinical PET procedures. But the optimal imaging conditions may vary depending on the image quality required, and shorter times may be sufficient for higher-sensitivity PET systems, such as semiconductor PET and other applications.

The current dynamic study did not include the patients with suspected metastatic lesions around pelvic organs to avoid the influence of gradual changes in bladder size during dynamic PET acquisition. Since the bladder size changes during PET imaging, peribladder lesions such as the uterus, ovaries and pararectal lymph nodes are expected to migrate. Therefore, repeated imaging may degrade PET image quality in the pelvic floor lesions. On the other hand, cases of large trunk movement during imaging are very rare. However, as shown in Fig. 3, upper extremity movement can cause attenuation correction errors in the pelvic region. Therefore, dynamic imaging may also be appropriate for cases of pelvic lesions. It may be necessary to consider whether cases suspected with pelvic lesion should be included in the future.

There are several methods for motion correction on PET/CT studies. Cardiac and respiratory motion may be rather easily corrected using ECG and/or a respiratory gating system [17–19]. Some studies have focused on altering CT transmission acquisition to incorporate the effects of respiratory and cardiac motion, including averaging cine acquisitions collected over one or more breath cycles and low-pitch CT protocols [20–23]. The use of cardiac attenuation correction-specific protocols resulted in a substantial reduction in misalignment, with results comparable to those of dedicated PET systems [24–26].

There are a several limitations in the study. First, the volume of image data is large, and, therefore, more time can be needed for reconstruction and data management than a conventional single scan. But the current system allows relatively rapid data management and also has enough space to store images for certain period. Next, we have used body weight corrected dose of FDG administration (3.0 MBq/kg). But we did not analyze the body weight for the current analysis.

Such whole-body dynamic imaging is not available in all PET systems. But since this acquisition is useful for reducing or eliminating artifacts caused by body movement, this elegant technique should be available at many PET centers in the near future.

In conclusion, serial dynamic whole-body FDG PET imaging can minimize body motion artifacts by summing only the images acquired before the body motion. Such serial dynamic study may be a choice for PET imaging to eliminate motion artifacts as much as possible.
